# Characteristics of MGUS and Multiple Myeloma According to the Target of Monoclonal Immunoglobulins, Glucosylsphingosine, or Epstein-Barr Virus EBNA-1

**DOI:** 10.3390/cancers12051254

**Published:** 2020-05-15

**Authors:** Adrien Bosseboeuf, Nicolas Mennesson, Sophie Allain-Maillet, Anne Tallet, Eric Piver, Olivier Decaux, Caroline Moreau, Philippe Moreau, Philippe Lehours, Francis Mégraud, Valéry Salle, Edith Bigot-Corbel, Jean Harb, Sylvie Hermouet

**Affiliations:** 1 Centre de Recherche en Cancérologie et Immunologie Nantes-Angers (CRCINA), Inserm, Université de Nantes, Université d’Angers, 44000 Nantes, France; adrien.bosseboeuf@gmail.com (A.B.); nicolas.mennesson@univ-nantes.fr (N.M.); sophie.allain@univ-nantes.fr (S.A.-M.); edith.bigot@univ-nantes.fr (E.B.-C.); jean.harb@univ-nantes.fr (J.H.); 2 Laboratoire de Biochimie, Centre Hospitalier Universitaire (CHU) Tours, 37000 Tours, France; anne.tallet@chu-tours.fr (A.T.); piver_e@univ-tours.fr (E.P.); 3 Inserm UMR966, 37000 Tours, France; 4 Internal Medicine, CHU Rennes, 35000 Rennes, France; olivier.decaux@chu-rennes.fr; 5 Laboratoire de Biochimie, CHU Rennes, 35000 Rennes, France; caroline.moreau@chu-rennes.fr; 6 Hematology Department, CHU Nantes, 44000 Nantes, France; philippe.moreau@chu-nantes.fr; 7 Laboratoire de Bactériologie, CHU Bordeaux, 33000 Bordeaux, France; philippe.lehours@u-bordeaux.fr (P.L.); francis.megraud@chu-bordeaux.fr (F.M.); 8 Inserm U1053, Université de Bordeaux, 33000 Bordeaux, France; 9 Médecine Interne et Maladies Systémiques, CHU Amiens, 80000 Amiens, France; Salle.Valery@chu-amiens.fr; 10 Laboratoire de Biochimie, CHU Nantes, 44000 Nantes, France; 11 Centre de Recherche en Transplantation et Immunologie (CRTI), UMR1064, Inserm, Université de Nantes, 44000 Nantes, France; 12 Laboratoire d’Hématologie, CHU Nantes, 44000 Nantes, France

**Keywords:** multiple myeloma, monoclonal gammopathy of undetermined significance (MGUS), monoclonal immunoglobulin, antigen specificity, auto-antigen, glucosylsphingosine (GlcSph), lysoglucosyl-ceramide (LGL1), sialylation, cytokine, inflammation, interleukin-13 (IL-13), interleukin-17 (IL-17), interleukin-26 (IL-26)

## Abstract

Chronic stimulation by infectious or self-antigens initiates subsets of monoclonal gammopathies of undetermined significance (MGUS), smoldering multiple myeloma (SMM), or multiple myeloma (MM). Recently, glucosylsphingosine (GlcSph) was reported to be the target of one third of monoclonal immunoglobulins (Igs). In this study of 233 patients (137 MGUS, 6 SMM, 90 MM), we analyzed the GlcSph-reactivity of monoclonal Igs and non-clonal Igs. The presence of GlcSph-reactive Igs in serum was unexpectedly frequent, detected for 103/233 (44.2%) patients. However, GlcSph was targeted by the patient’s monoclonal Ig for only 37 patients (15.9%); for other patients (44 MGUS, 22 MM), the GlcSph-reactive Igs were non-clonal. Then, the characteristics of patients were examined: compared to MM with an Epstein-Barr virus EBNA-1-reactive monoclonal Ig, MM patients with a GlcSph-reactive monoclonal Ig had a mild presentation. The inflammation profiles of patients were similar except for moderately elevated levels of 4 cytokines for patients with GlcSph-reactive Igs. In summary, our study highlights the importance of analyzing clonal Igs separately from non-clonal Igs and shows that, if autoimmune responses to GlcSph are frequent in MGUS/SMM and MM, GlcSph presumably represents the initial pathogenic event for ~16% cases. Importantly, GlcSph-initiated MM appears to be a mild form of MM disease.

## 1. Introduction

Multiple myeloma (MM) is preceded by an asymptomatic stage termed monoclonal gammopathy of undetermined significance (MGUS), eventually followed by an intermediate stage called smoldering multiple myeloma (SMM). In all cases, a plasmacytic clone expands and produces large quantities of a single immunoglobulin (Ig), termed “monoclonal Ig”. Following the acquisition of genetic alterations in clonal plasma cells, a minority of MGUS eventually progresses over the years towards SMM, then overt MM [1,2,3,4]. The causes of MGUS have long remained unknown, but recent studies of the antigen specificity of monoclonal Igs indicate that chronic antigenic stimulation by an infectious pathogen or by a self-antigen, notably glucosylsphingosine (GlcSph)—also called lysoglucosylceramide (LGL1)—appears to be a frequent pathogenic mechanism involved both in sporadic MGUS and MM and in monoclonal gammopathies associated with Gaucher disease (GD) [5,6,7,8,9]. In GD, germline mutations in the glucocerebrosidase (*GBA*) gene result in the accumulation of glucocerebroside and GlcSph. GD patients may present with various clinical manifestations and complications, notably an increased risk of developing MGUS and MM [10]. Nair et al. recently showed that GlcSph is a frequent target of monoclonal Igs of GD-associated MGUS and MM [8,9]. These findings are of major importance since, for the first time, treatments that aim to suppress the target of the monoclonal Ig can be envisioned for MGUS patients, thus offering the possibility to prevent evolution toward SMM and MM. Indeed, for GD patients with a MGUS, eliglustat therapy aimed at reducing their level of GlcSph, the immunogenic lipid target of GD monoclonal Igs, successfully reduced the amount of monoclonal Ig [11]. Target antigen reduction therapy could also improve the response of MM patients to classic chemotherapy, as observed with antiviral treatment for MGUS and MM patients who presented with a monoclonal Ig specific for hepatitis C virus (HCV) [12,13].

According to present knowledge, one-third of patients with sporadic MGUS and MM may have a monoclonal Ig specific for an infectious pathogen, implying chronic latent infection, and another third may have a monoclonal Ig specific for GlcSph, consistent with chronic autoimmunity [5,8]. However, in these studies the technical approaches differed, which prompted us to analyze, in parallel, the GlcSph—or infectious pathogen—specificity of the purified monoclonal Ig and of non-clonal Igs from patients diagnosed with sporadic MGUS, SMM, or MM. Polyclonal Igs obtained from serum samples of donors without blood disease and of matching ages (≥60) served as controls. We report that auto-antibodies specific for GlcSph were detected in the serum of 49.3% MGUS/SMM and 38.9% MM patients, and that GlcSph was targeted by the monoclonal Ig in 16.8% MGUS/SMM and 14.4% MM cases. Thus, for the majority of MGUS/SMM and MM patients with GlcSph-reactive Igs in serum, these Igs were non-clonal. Importantly, MM patients who presented with a GlcSph-reactive monoclonal Ig appeared to have a mild form of MM disease. We also report on the inflammation status of patients with GlcSph-specific Ig(s) and on the level of sialylation of GlcSph-specific monoclonal IgGs.

## 2. Results

### 2.1. Patient Characteristics

In this retrospective study, serum samples were collected from 233 patients with a monoclonal Ig (137 MGUS, 6 SMM, 90 MM) and 46 healthy donors (without blood disease and of age ≥60). For MGUS patients, the monoclonal Ig was an IgG for 129 cases, and an IgA for 8 cases. SMM cases were all IgG SMM. For MM patients, the monoclonal Ig was an IgG for 75 cases, and an IgA for 15 cases. Table S1 shows that the median age of patients at the time of diagnosis was 68.1 years for MGUS/SMM patients and 67.0 years for MM patients. The male ratio was 57.1% for MGUS/SMM and 54.5% for MM. The median quantity of monoclonal Ig at the time of diagnosis was 16.0 g/L for MGUS/SMM patients and 23.0 g/L for MM patients. Fifty-nine (68.6%) MM patients presented with bone lesions. In the MM cohort, 28.0% of patients presented with International Staging System (ISS) stage III at the time of diagnosis, and 43.8% had Durie-Salmon Staging (DSS) stage III.

Because the quantity of serum available varied depending on patients, not all the assays used in this study (GlcSph assay, MIAA assay, sialylation studies, cytokine quantification) could be performed for all patients. Information on the number of patients analyzed with each assay is provided in Figure S1.

### 2.2. Specificity of Recognition of Purified Monoclonal Igs

**Figure 1:**
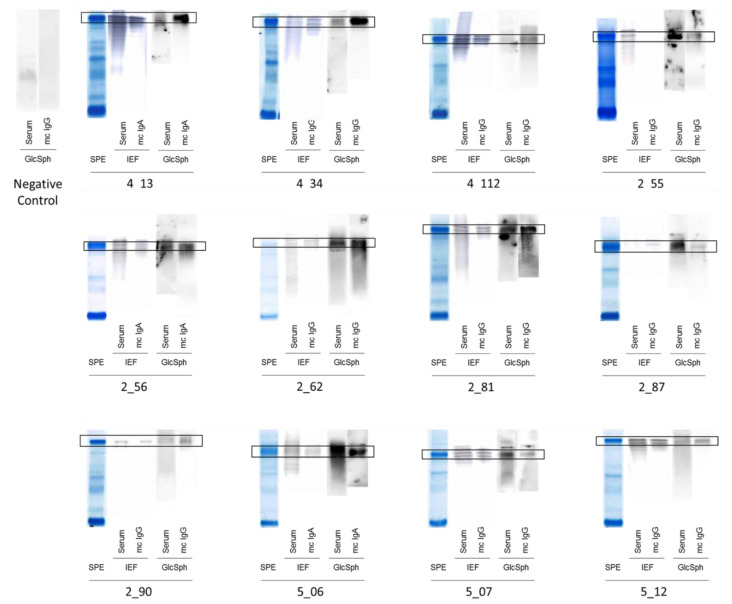
Glucosylsphingosine (GlcSph)-reactivity of serum Igs and monoclonal Igs obtained for patients with monoclonal gammopathy of undetermined significance (MGUS). Measurement of Ig concentration, separation of monoclonal Igs from other Igs, and verification of purity were performed as published [5,6,7,14,15]. Purification starts with the separation of serum proteins with high resolution agarose gel electrophoresis (SAS-MX high resolution, Helena Biosciences, Gateshead, UK). Then, the monoclonal Ig is cut from the gel and eluted in phosphate buffer saline (PBS). The purity of the monoclonal Ig preparation is verified by isoelectric focusing (IEF) on an agarose gel (pH 3–10) followed by blotting and immuno-revelation by an anti-human IgGγ chain or anti-IgAα chain antibody labeled with peroxidase. For GlcSph-specific immunoblotting, polyvinylidene fluoride (PVDF) membranes are incubated for 90 min in 100 μg/mL of GlcSph in 0.1 M sodium bicarbonate, rinsed in PBS and 0.1% Tween 20 detergent, and then blocked with 5% bovine serum albumin (BSA) in PBS and 0.1% Tween 20. Samples of serum and purified monoclonal Ig are submitted to agarose gel electrophoresis, and then the gels are blotted onto the GlcSph-saturated membranes by diffusion blotting during 12 min. After blocking with 2.5% BSA in PBS and 0.1% Tween 20, membranes are incubated with anti-human IgG or IgA horseradish peroxidase (HRP)-conjugated secondary antibody, washed, and revealed by chemiluminescence. Signals corresponding to the patient’s monoclonal Ig are encircled in black. The negative control is a patient with no GlcSph-reactive Ig in serum. SPE = Serum protein electrophoresis; Mc Ig = purified monoclonal Ig. The GlcSph-reactivity of serum Igs and monoclonal Igs obtained for 12 other MGUS patients are shown in Figure S2.

**Figure 2:**
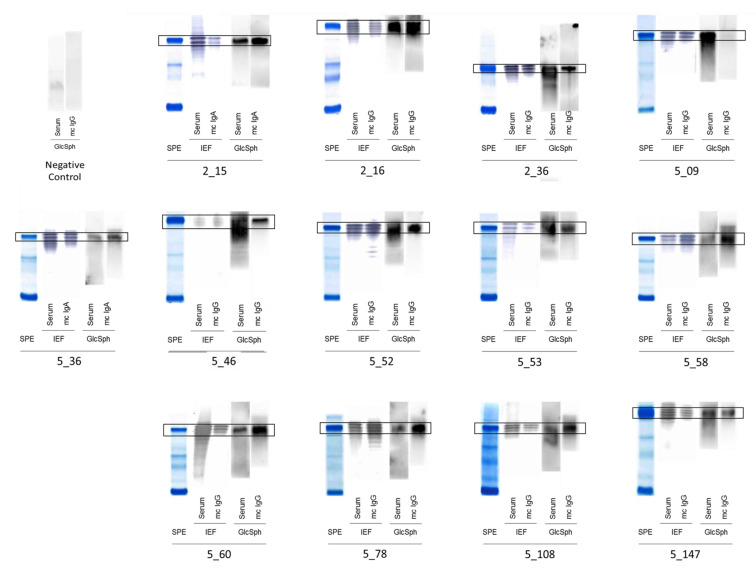
GlcSph-reactivity of serum Igs and monoclonal Igs in MM. Measurement of Ig concentration, separation of monoclonal Igs from other Igs, and verification of purity are performed as described above (Figure 1), in Methods, and published previously [5,6,7,14,15]. SPE = Serum protein electrophoresis; Mc Ig = purified monoclonal Ig. Signals corresponding to the patient’s monoclonal Ig are encircled. The negative control is a patient with no GlcSph-reactive Ig in serum.

For each patient, the monoclonal Ig was separated from polyclonal (non-clonal) Igs present in the blood serum and purified individually, and then the purity of the monoclonal Ig preparation was verified using isoelectric focusing (IEF), as previously described [5,6,7,14,15]. The antigenic specificity of each purified monoclonal Ig was analyzed using a home-made GlcSph (LGL1) immunoblot assay adapted from Nair et al. [8,9] and the multiplexed infectious antigen microarray (MIAA) assay [5,14,15]. For both the GlcSph and MIAA assays, blood serum (containing polyclonal Igs in addition to the patient’s monoclonal Ig) and the purified monoclonal Ig preparation were analyzed in parallel. The presence of GlcSph-reactive Igs in serum was unexpectedly frequent, observed for 68/143 MGUS/ SMM patients (63/137 MGUS, 5/6 SMM) and for 35/90 MM patients (Table 1, Figure 1 and Figure 2, Figures S2 and S3).

When the purified monoclonal Igs from MGUS/SMM or MM patients were analyzed (Table 1, Figure 1, Figure 2 and Figure S2), 24/143 (16.8%) MGUS/SMM patients (24 MGUS, 0 SMM) and 13/90 (14.4%) of MM patients had a purified monoclonal Ig that recognized GlcSph. Among the 41 control donors of similar age and without blood disease who could be tested for GlcSph-reactive Igs, 39/41 (95.1%) were negative (Figure S3). For 2 control donors, the search for SphGlc-reactive Igs in serum was positive; these individuals did not present any monoclonal or oligoclonal Igs (Figure S3).

In parallel, the reactivity of the purified monoclonal Ig from all patients was tested against nine infectious pathogens using the MIAA assay [5,14,15]. There was no cross-reactivity with infectious pathogens for GlcSph-reactive monoclonal Igs. The monoclonal Ig from 88/143 (61.5%) MGUS/SMM patients and 29/90 (32.2%) MM patients specifically targeted a single pathogen of the multiplexed infectious antigen micro-array (MIAA) assay (Table 1). As published, the most frequent infectious target of monoclonal Igs of MGUS/SMM and MM patients was Epstein-Barr virus (EBV) nuclear antigen-1 (EBNA-1), recognized by 75/233 (32.2%) monoclonal Igs, and the frequency was similar in MGUS/SMM and in MM [5]. In these cohorts, MGUS patients were significantly more likely to have a monoclonal Ig reactive against infectious pathogens other than EBV than MM patients. The infectious pathogens recognized by monoclonal Igs included herpes simplex virus 1 (HSV-1) (*n* = 15), varicella zoster virus (VZV) (*n* = 9), and cytomegalovirus (CMV) (*n* = 5). Altogether, we were able to determine the target of the monoclonal Ig (GlcSph or infectious pathogen) for 112/143 (78.3%) MGUS/SMM patients and 42/90 (46.7%) MM patients. The percentage of monoclonal Igs with an identified target was significantly higher for MGUS/SMM patients than for MM patients (*p* < 0.00001, Fisher exact test) (Table 1).

**Figure 3:**
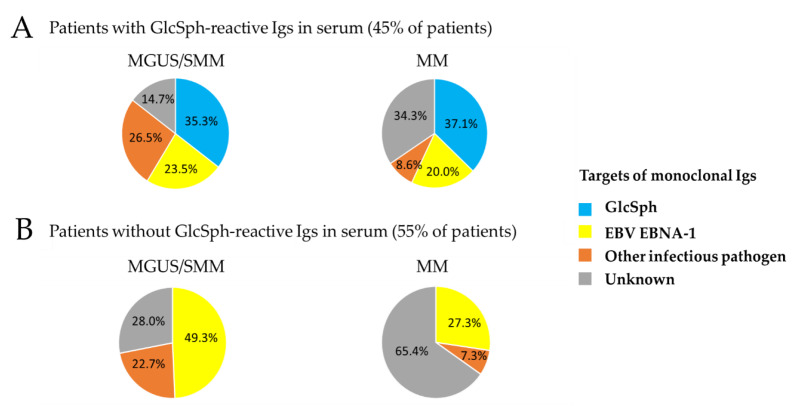
Identified targets of monoclonal Igs in MGUS/SMM and MM, according to the presence or absence of GlcSph-reactive Igs. Representation of the percentage (%) of patients with a monoclonal Ig that targets GlcSph, EBV EBNA-1, or another MIAA pathogen. “Unknown”: patients for whom the target of the monoclonal Ig has not been identified (unknown target). (**A**) Targets of monoclonal Igs from patients with GlcSph-reactive Igs (clonal or non-clonal). (**B**) Targets of monoclonal Igs from patients who do not have GlcSph-reactive Igs.

We then investigated whether the specificity of a patient’s monoclonal Ig may differ depending on the existence of a non-clonal autoimmune response against GlcSph (i.e., presence of non-clonal GlcSph-reactive Igs in serum) (Table 2, Figure 3). In the MGUS/SMM cohort, patients with non-clonal GlcSph-reactive Igs were significantly more likely to have a monoclonal Ig specific for an infectious pathogen other than EBV (40.9%) than patients without GlcSph-reactive Igs (22.7%, *p* = 0.0398). In the MM cohort, no difference was observed, and the rate of monoclonal Igs with no known target were similarly high (54.5% for MM with GlcSph-reactive Igs, and 65.4% without).

### 2.3. Characteristics of MGUS/SMM Patients with GlcSph-Reactive Igs

Little information was available for MGUS patients so for the MGUS/SMM cohort we were able to analyze the sex ratio, age, and quantity of monoclonal Ig at diagnosis (Table S2). Compared to MGUS/SMM patients without GlcSph-reactive Igs, patients with GlcSph-reactive Igs (63 MGUS, 5 SMM) presented with a lower amount of monoclonal Ig (median: 13.0 g/L vs. 17.2 g/L, *p* = 0.0123, Mann–Whitney test). There was no significant difference in age or sex ratio between the two groups of patients. We then analyzed the group of 24 MGUS patients with a GlcSph-reactive monoclonal Ig and found no significant difference in sex ratio, age, or amount of monoclonal Ig, compared to the group of MGUS/SMM patients with a monoclonal Ig specific for EBV EBNA-1. As published previously, MGUS/SMM patients with an EBNA-1-specific monoclonal Ig (51 MGUS, 2 SMM) were more frequently male (69.4% vs. 49.4%, *p* = 0.0294, Fisher exact test) [5].

### 2.4. Characteristics of MM Patients with GlcSph-Reactive Igs

Complete biological information was not available for all MM patients; therefore, the number of patients with data varies depending on the biological parameter studied (Table 3). Between groups of MM patients who presented with or without GlcSph-reactive Igs (non-clonal or monoclonal) in serum, no significant difference was found in sex ratio, age at the time of diagnosis, amount of monoclonal Ig, presence of bone lesions, β_2_-microglobulin, ISS or DSS scores, calcemia, creatinin, or blood counts (leukocytes, platelets, red blood cells, hemoglobin level).

We then compared the characteristics of MM patients with a GlcSph-reactive monoclonal Ig to those of MM patients with a monoclonal Ig specific for EBV EBNA-1. MM patients with a GlcSph-reactive monoclonal Ig were significantly less likely to present with bone lesions or DSS stage III (severe disease) than MM patients with an EBV EBNA-1-specific monoclonal Ig. A similar tendency (less bone lesions, less DSS stage 3) was noted compared to MM patients with a monoclonal Ig of undetermined specificity, but differences were not significant.

### 2.5. Sialylation Status of GlcSph-Reactive Igs from MGUS, SMM and MM Patients

**Figure 4:**
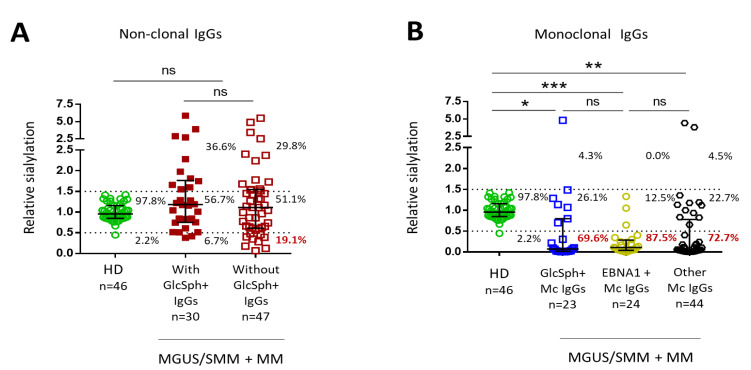
Level of sialylation of IgGs from MGUS/SMM and MM patients. Sialylation studies were performed using an ELLA (Enzyme Linked Lectin Assay) for IgG sialylation detection, as well as an ELISA (Enzyme Linked Immuno Sorbent Assay) for total IgG detection [15]. Clonal and non-clonal IgG fractions were prepared as described in Methods and published [15]. The relative sialylation of IgGs was expressed as the sialic acid/IgG optic density (OD) ratio. (**A**) Degree of sialylation of polyclonal IgGs from 43 healthy donors (HD) (OD ratio between 0.5 and 1.5) and of non-clonal IgGs from 77 patients. (**B**) Degree of sialylation of 91 purified monoclonal IgGs analyzed according to the target of the monoclonal IgG. Bars indicate means ± SEM. The percentages indicate the % of IgGs with a low (OD ratio < 0.5), normal (OD ratio: 0.5–1.5) or high (OD ratio > 0.5) degree of sialylation. Statistical analysis was performed using one-way ANOVA test followed by Tukey’s multiple comparison test. * *p* < 0.05, ** *p* < 0.01, *** *p* < 0.001. NS: not significant.

The degree of sialylation of the Fc fragments of Igs was studied in parallel in samples of purified non-clonal Igs and in samples of purified monoclonal Igs, as published previously [15]. Sialylation studies were performed for 91 patients (30 MGUS, 4 SMM, 57 MM) (Figure S1) and 46 healthy donors. Since our cohorts included few patients with a monoclonal IgA, sialylation studies were only performed for IgGs; data are presented in Figure 4. Because of insufficient material for certain patients, and also because certain MM patients no longer produced enough non-clonal IgGs, unseparated serum Igs were studied for 77/91 patients.

The level of sialylation of non-clonal IgGs for the majority of patients with or without GlcSph-reactive IgGs was normal (OD ratio between 0.5 and 1.5, as observed for healthy donors) (Figure 4A). A minority of patients had hypersialylated IgGs (OD ratio >1.5), but there was no significant difference between groups of patients with or without GlcSph-reactive IgGs. Non-clonal IgGs from patients with GlcSph-reactive IgGs tended to have more frequently normal or increased sialylation (OD ratio ≥0.5 for 28/30 (93.3%) patients) than non-clonal IgGs from patients without GlcSph-reactive IgGs (OD ratio ≥0.5 for 38/47 (80.8%) patients) but the difference was not significant (*p* = 0.1857, Fisher exact test). As published [15], purified monoclonal IgGs were significantly less sialylated than polyclonal IgGs from healthy donors, but there was no difference in level of sialylation according to the target of the monoclonal IgG (GlcSph, EBNA-1, other) (Figure 4B).

### 2.6. Inflammation Status of MGUS, SMM, and MM Patients with LGL1-Reactive Igs

**Figure 5:**
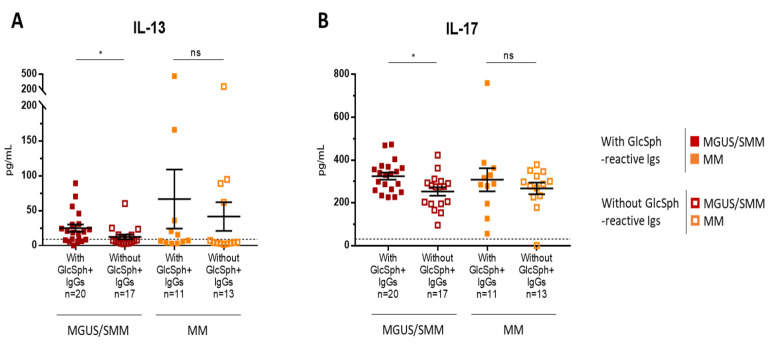
Differences in cytokine levels according to the presence of GlcSph-reactive polyclonal Ig(s) for MGUS/ SMM and MM patients. Forty-two cytokines, chemokines and receptors were quantified using the Luminex technology and Bio-Plex Pro Human Cytokine Panel kits [15]. The levels of only 2 cytokines, IL-13 (**A**) and IL-17 (**B**), were significantly different depending on the presence of polyclonal GlcSph-reactive IgGs. Bars indicate means + SEM; note that each figure has a different scale. GlcSph+: with GlcSph-reactive IgG(s) in serum. MGUS/SMM patients with (■) or without (☐) GlcSph-reactive IgGs in serum; MM patients with (■) or without (☐) GlcSph-reactive IgGs in serum. (*) *p* < 0.05, Mann–Whitney *t*-test. The dotted line represents the maximal normal value observed in healthy individuals. NS: not significant.

Our previous studies showed that, out of 42 cytokines, chemokines, and receptors linked to inflammation measured in serum using Luminex technology and Bio-Plex Pro human cytokine panel kits (Bio-Rad, Hercules, CA, USA), 26 were elevated in MGUS, SMM, and MM compared to healthy individuals [15]. A list of the 42 molecules linked to inflammation measured in the serum of patients, with the range of values observed in healthy controls, is provided in Table S2. Quantitative analysis of the 42 molecules was performed for 61 patients (33 MGUS, 4 SMM, 24 MM) (see Figure S1). All 61 patients had a monoclonal IgG. In the MGUS/SMM group, the levels of only 2/42 molecules, anti-inflammatory interleukin-13 (IL-13) and pro-inflammatory IL-17, were moderately but significantly elevated for patients with GlcSph-reactive IgGs (IL-13 (pg/mL): *n* = 20, median, 18.5; range, 0.01–89.02; IL-17 (pg/mL): *n* = 20, median, 315.2; range 226–472) than for MGUS/ SMM patients without GlcSph-reactive IgGs (IL-13 (pg/mL): *n* = 17, median, 7.69; range, 2.4–60.1; IL-17 (pg/mL): *n* = 17, median, 274.7; range, 96–423) (Figure 5A,B). Of note, IL-17 has been shown to be elevated in auto-immune diseases. For patients with MM, however, there was no significant difference in cytokine or chemokine levels between patients with or without GlcSph-reactive IgGs (Figure 5A,B).

**Figure 6:**
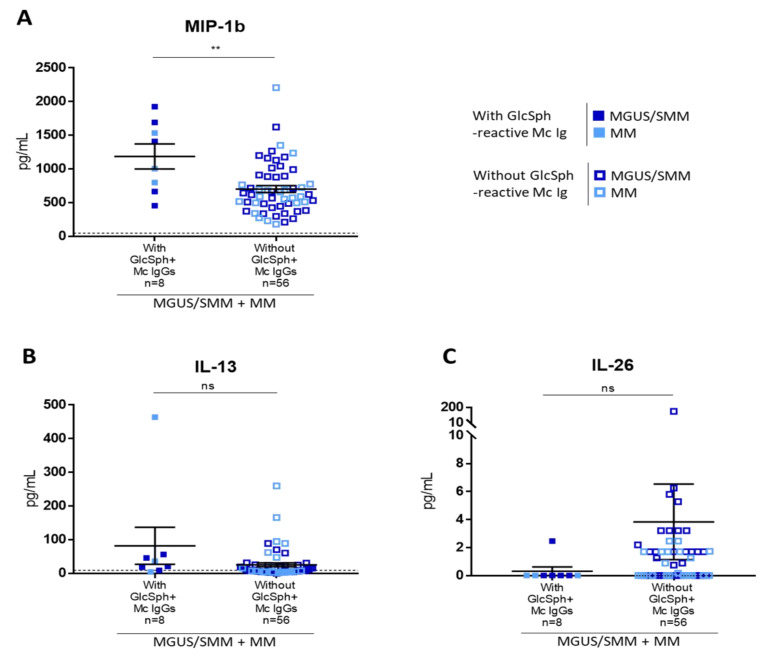
Differences in cytokine levels according to the presence of a GlcSph-reactive monoclonal Ig for MGUS/SMM and MM patients. The levels of MIP-1β (**A**) were significantly different depending on the presence of a monoclonal GlcSph-reactive IgG, whereas the levels of IL-13 (**B**) and IL-26 (**C**) were not. Each figure has a different scale. Bars indicate means + SEM. MGUS/SMM (■) and MM (■) patients with a GlcSph-reactive monoclonal (Mc) IgG; MGUS/SMM (☐) and MM (☐) patients without a GlcSph-reactive Mc IgG. (**) *p* < 0.01, Mann–Whitney *t*-test. The dotted line represents the maximal normal value observed in healthy individuals. NS: not significant.

We then studied patients who presented with a GlcSph-reactive monoclonal IgG. Due to the small number of patients with GlcSph-reactive monoclonal IgG (*n* = 8), for this analysis MGUS and MM patients were studied together. Chemokine macrophage inflammatory protein β1 (MIP-1β, or CCL4) was the only molecule present at a significantly higher level in the serum of patients with a monoclonal Ig that targeted GlcSph (*n* = 8, median, 1207 pg/mL; range, 456–1926 pg/mL), compared to other patients (*n* = 56, median, 617.9 pg/mL; range, 180.2–2207.5 pg/mL) (Figure 6A). A tendency to increased IL-13 (*n* = 8, median, 28.5 pg/mL; range, 4.3–464 pg/mL) and low IL-26 (*n* = 8, median, 0.01 pg/mL; range, 0.01–2.47 pg/mL) was also observed for patients with a GlcSph-reactive monoclonal Ig, compared to other patients (IL-13: *n* = 56, median, 7.7 pg/mL; range, 0.01–259.8 pg/mL; IL-26: *n* = 56, median, 0.47 pg/mL; range, 0.01–151.6 pg/mL, *n* = 56), but differences were not significant (Figure 6B,C). There was no difference in IL-17 levels.

**Figure 7:**
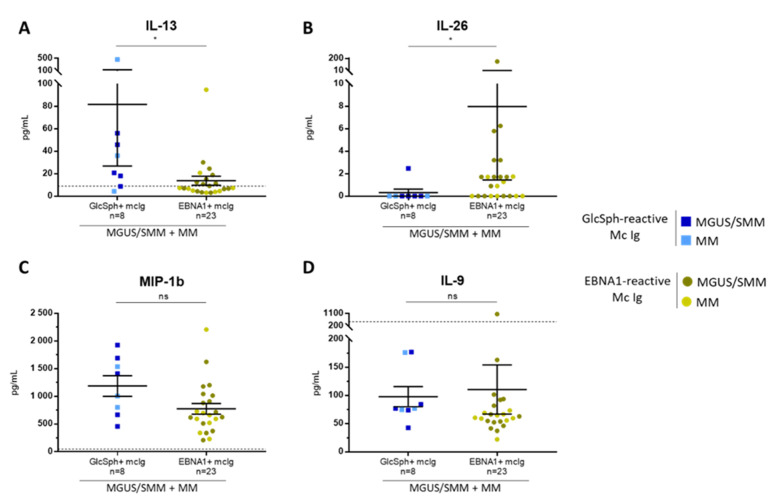
Differences in cytokine levels according to the presence of GlcSph- or EBV EBNA-1-reactive monoclonal Ig for MGUS/SMM and MM patients. The levels of 2 cytokines, IL-13 (**A**) and IL-26 (**B**) were significantly different depending on the antigen specificity of the monoclonal Ig, whereas the levels of MIP-1β (**C**) and IL-9 (**D**) were not. Note that each figure has a different scale; bars indicate means ± SEM. GlcSph+: with GlcSph-reactive monoclonal (mc) IgG; EBNA-1+: with EBV EBNA-1-reactive mc IgG. (■) MGUS/SMM and (■) MM patients with GlcSph-reactive mc IgG; (⬤) MGUS/SMM and (⬤) MM patients with an EBNA-1-reactive mc IgG. (*) *p* < 0.05, Mann–Whitney *t*-test. The dotted line represents the highest normal value observed in healthy donors. NS: not significant.

The levels of cytokines were also analyzed according to the specificity of recognition of the patient’s monoclonal Ig (GlcSph vs. EBV EBNA-1) (Figure 7). IL-13 was significantly higher (*n* = 8, median, 28.5 pg/mL; range, 4.3–464 pg/mL) and IL-26 significantly lower (*n* = 8, median, 0.01 pg/mL; range, 0.01–2.5 pg/mL) for patients with a GlcSph-reactive monoclonal IgG compared to patients with an EBNA-1-reactive monoclonal IgG (IL-13: *n* = 23, median, 7.3 pg/mL; range, 3.03–94.8 pg/mL; IL-26: *n* = 23, median, 1.3 pg/mL; range, 0.01–151.6 pg/mL) (Figure 7A,B). For patients with a GlcSph-reactive monoclonal IgG, a tendency toward higher levels of MIP-1β and IL-9 (MIP-1β: *n* = 8, median, 1207 pg/mL; range, 456–1926 pg/mL; IL-9: *n* = 8, median, 76.6 pg/mL; range, 42.3–177 pg/mL) was also observed, compared to patients with an EBV EBNA-1-reactive monoclonal IgG (MIP-1β: *n* = 23, median, 659 pg/mL; range, 210–2207 pg/mL; IL-9: *n* = 23, median, 62.7 pg/mL; range, 22.2–1063 pg/mL) but differences were not significant (Figure 7C,D). No difference in IL-17 was observed.

In summary, the presence of GlcSph-reactive IgG(s) was associated with modest increases in the levels of only 4 cytokines (anti-inflammatory IL-13, pro-inflammatory IL-17 and MIP-1β, and immuno-stimulant IL-9), while the level of anti-microbial IL-26 was very low.

## 3. Discussion

Our study confirms the frequent presence of GlcSph-reactive Igs in the blood serum of patients diagnosed with sporadic MGUS and MM (without GD), as first reported by Nair et al. [8]. However, working with purified monoclonal Igs allowed us to distinguish between patients with clonal or non-clonal GlcSph-reactive Ig(s), and we show that GlcSph is targeted by the patient’s clonal Ig in 16% of MGUS and MM cases with GlcSph-reactive Igs, or about half the fraction (33%) reported by Nair et al., whose studies did not separate clonal from non-clonal Igs [8]. Thus, our study highlights the importance, in the context of MGUS and MM, of studying the monoclonal Igs of patients separately from other, non-clonal Igs. Our data also imply that chronic stimulation by self-antigen GlcSph likely underlies the initiation of sporadic MGUS and MM in ~16% cases.

In our cohorts, ~45% of patients with sporadic MGUS or MM presented with GlcSph-reactive Igs in serum, and 35.9% of these patients had a monoclonal Ig that targeted GlcSph. For patients who presented with non-clonal GlcSph-reactive Igs, the monoclonal Ig targeted an infectious pathogen (50% cases in MGUS, 28.6% in MM), or the target of the monoclonal Ig was not identified (14.7% in MGUS, 34.3% in MM), in a manner very similar to patients who do not have GlcSph-reactive Igs.

The high frequency of non-clonal GlcSph-reactive Igs observed in sporadic MGUS and MM was unexpected and quite intriguing. In the context of GD, the presence of polyclonal GlcSph-reactive autoantibodies may be interpreted as an effort to counter the high blood level of GlcSph characteristic of carriers of biallelic germinal mutations in the glucocerebrosidase (*GBA*) gene. Yet non-clonal GlcSph-reactive autoantibodies have been reported in acquired diseases, particularly in auto-immune diseases (in the absence of GD). A recent study of 140 patients diagnosed with 12 autoimmune diseases, including rheumatoid arthritis (35 patients), psoriatic arthritis (20 patients), systemic lupus erythematosus (20 patients), and multiple sclerosis (2 patients) found that 26/140 (19%) of these patients carried non-clonal GlcSph-reactive autoantibodies [16]. These authors proposed that the presence of polyclonal GlcSph-reactive Igs may reflect increased B-cell activation and proliferation, cytokine stimulation, or/and a general predisposition towards autoimmunity [16].

In regard to cytokine stimulation, it is well established that both MGUS patients and MM patients, with or without treatment, present with strong and chronic inflammation [15,17,18,19,20,21]. However, we found very few differences in the inflammation profiles of patients with or without non-clonal GlcSph-reactive Igs. Among 42 cytokines measured, significant changes were observed for only 4 cytokines (IL-13, IL-17, IL-26, MIP-1β), three of which were reported as overexpressed in auto-immune diseases (IL-17, IL-13, IL-26). Pro-inflammatory IL-17 was significantly elevated in patients with GlcSph-reactive Igs but only for MGUS patients. Interestingly, IL-17 is increased in auto-immune diseases and is also known to decrease the expression of α2,6-sialyltransferase, which contributes to lower IgG sialylation in auto-immune diseases [22,23,24,25]. Accordingly, we previously reported an inverse correlation between levels of IL-17 and the degree of sialylation of IgGs in both MGUS and MM [15]. Inversely, the anti-inflammatory IL-13 was positively correlated with the sialylation of monoclonal IgGs [15]. In the present study, patients with a GlcSph-reactive monoclonal IgG had higher levels of IL-13 than patients with an anti-EBV EBNA-1 monoclonal IgG. In contrast, the level of IL-26, a pro-inflammatory, anti-microbial cytokine reported to be elevated in inflammatory arthritis, was low for patients with a GlcSph-reactive monoclonal IgG [26]. Consistent with the actions of IL-17 and IL-13 and their elevated levels in patients with GlcSph-reactive IgGs, hyposialylation of non-clonal IgGs was observed for these patients (6.7% vs. 19.2% of patients without GlcSph-reactive IgGs, difference not significant). Finally, there was a tendency for patients with a GlcSph-reactive monoclonal IgG towards a higher level of IL-9, a cytokine that promotes immune responses and is elevated in many inflammatory diseases [27,28].

Importantly, GlcSph itself is pro-inflammatory, and chronic lipid-mediated inflammation is thought to facilitate the development of a variety of chronic diseases [29,30,31]. Chronic inflammation is a characteristic shared by the different categories of patients who acquire GlcSph-reactive Igs: sporadic MGUS and MM, Gaucher disease, and patients with chronic inflammatory diseases, including auto-immune diseases. We propose that inflammation may underlie the non-clonal anti-GlcSph autoimmune process observed in these patients, particularly patients with sporadic MGUS and MM whose monoclonal Ig targets an antigen other than GlcSph. For patients with a GlcSph-specific monoclonal Ig, chronic inflammation may also underlie the anti-GlcSph autoimmune process, first as non-clonal autoimmune response; then, over time, the autoimmune process may become oligoclonal, then monoclonal (i.e., reach the MGUS stage). Thus, polyclonal GlcSph-reactivity may serve as a precursor to the emergence of monoclonality, a pathogenic process observed in other cohorts that may be underestimated in MM due to the frequent suppression of non-clonal Igs [9,32]. In this retrospective study, patient DNA was not available for genetic studies; therefore, the possibility of a “latent”, low grade, or atypic form of GD associated with sporadic cases of MGUS and MM with GlcSph-reactive monoclonal Ig could not be addressed.

This study has limitations. Ideally, the characteristics of monoclonal Ig observed in vitro would have been verified by direct evaluation of the antigen reactivity of clonal BCRs. Unfortunately, this could not be done since we did not have access to patient plasma cells. The other limitations are the small size of the cohorts and limited clinical data. Obviously, studies of larger cohorts of MM patients are required to fully characterize GlcSph- or EBV EBNA-1-associated MM disease, but the already present data suggest that having a GlcSph-reactive monoclonal Ig could be of better prognosis than MM with an EBNA-1-reactive monoclonal Ig. This finding is consistent with Nair et al.’s initial report that MM patients with GlcSph-reactive Igs tended to have mild form of MM disease [8]. In our study, bone lesions were less frequent in MM patients with GlcSph-reactive monoclonal Ig, who typically presented with mild disease, since >80% had a I-II DSS score. In contrast, MM patients with EBV EBNA-1-specific monoclonal Ig presented with more severe disease [5]. Altogether, identification of the target of monoclonal Igs seems to matter and may become a new prognostic marker.

Last but not least, the target of monoclonal Igs may also matter in terms of treatment, since therapy aiming at reducing the target of a patient’s monoclonal Ig may be proposed to MGUS and MM patients [9,33]. In this regard, recent reports described the beneficial effect, in murine models and for GD patients who presented a GlcSph-reactive monoclonal Ig, of treatments that reduced the level of immunogenic glucolipid: a clear reduction of the amount of the monoclonal Ig was obtained for two patients [11,34]. Similarly, we and others reported that successful antiviral treatment benefited not only MGUS patients but also MM patients who produced a monoclonal Ig that targeted hepatitis C virus [12,13].

## 4. Materials and Methods

### 4.1. Patients

The study was promoted by the University Hospital of Nantes (# RC12 0085) with the approval of the local ethical committee and the Commission Nationale de l’Informatique et des Libertés (CNIL #912335). We examined 233 patients with a monoclonal Ig (137 MGUS, 6 SM, 90 MM) at the time of diagnosis at the Centres Hospitaliers Universitaires (CHUs) of Tours, Rennes, Nantes, Bordeaux, and Amiens (France) over the 2010–2016 period. Serum samples from 46 individuals of age ≥60 years without blood disease were also examined (Cryopep, Montpellier, France, and Etablissement Français du Sang (EFS) Pays de la Loire). Written informed consents were obtained from patients in the relevant clinical departments, as well as in the blood bank for healthy volunteers enrolled by the EFS Pays de La Loire. A convention has been signed between CRCINA, Inserm UMR1232 and CRTI, Inserm UMR1064, and EFS Pays de La Loire.

### 4.2. Separation of Monoclonal and Non-Clonal Igs

After clotting, blood samples were centrifuged at 2200× *g* (4 °C) and serum aliquots were frozen at −20 °C and at −80 °C. Concentrations of IgG, IgA, and IgM in serum were measured with an immuno-nephelemetric assay performed on a Beckman Immage Analyzer (Beckman Coulter, Villepinte, France). The concentration of the monoclonal (component) IgG is estimated by integrating the electrophoretic peak according to the orthogonal mode (the so-called “baseline method”). Purification of monoclonal Igs and verification of their purity have been described previously [5,6,7,14,15]. After separation using electric charge on agarose gel electrophoresis (SAS-MX high resolution, Helena Biosciences, Gateshead, UK), bands corresponding to monoclonal Igs were carefully cut, and proteins were eluted from gels into PBS. Non-clonal IgGs were similarly extracted from the immunoglobulin part of each lane, as far as possible from the monoclonal band, and then eluted from gels into PBS. Concentration of the purified monoclonal Igs or of non-clonal Igs was determined using the Nanodrop Spectro-photometer ND-1000 (ThermoFisher Scientific, Waltham, MA, USA) with the IgG extinction coefficient (ε = 1.36 for a solution of 1 mg/mL). The recovered Ig amount after purification varied from 40 to 70%, depending on experiments and the initial Ig concentration in serum. Purity of each monoclonal Ig fraction was analyzed by isoelectrophoresis and immunoblotting (homemade isoelectrofocusing (IEF) gel using a range of pH 3–10, blotting onto PVDF membrane and revelation using an horse-radish peroxidase (HRP)-labelled anti-human IgGγ chain for IgGs, and anti-human IgAα chain for IgAs (Dako, Glostrup, Denmark) [5,15]. Only highly purified monoclonal Igs were used for the studies below. This protocol of purification of monoclonal Igs has been validated previously with the verification of the purity of monoclonal Ig preparations for 10 patients using mass spectrometry [5,15]. Information on the number of patients whose monoclonal Ig and serum were analyzed using each type of assay is provided in Figure S1.

### 4.3. Analysis of the Specificity of Recognition of Monoclonal Igs

#### 4.3.1. The GlcSph (LGL1) Assay

Analysis of the presence of polyclonal or monoclonal Ig specific for glucosylsphingosine (GlcSph, or LGL1) was performed using an immunoblotting assay adapted from Nair et al. [8]. GlcSph (ref. 2086, with purity assessed at >98% by thin-layer chromatography) was purchased from Matreya LLC/Cayman Chemical (Ann Arbor, MI, USA) and stored frozen (at −20 °C) at a concentration of 20 mg/mL in ethanol as storage stock. Serum (containing polyclonal or non-clonal Igs ± patient’s monoclonal Ig) and purified monoclonal IgGs and IgAs were systematically studied in parallel. Polyvinylidene fluoride (PVDF) membranes were incubated for 90 min in 100 μg/mL of GlcSph in 0.1 M sodium bicarbonate, rinsed 3 times in PBS and 0.1% Tween 20 detergent, and then blocked for 2 h with 5% bovine serum albumin (BSA) in PBS and 0.1% Tween 20. Samples of serum or purified monoclonal IgG or IgAs were submitted to agarose gel electrophoresis, and then the gels were blotted onto the GlcSph-saturated membranes by diffusion blotting during 12 min [35,36]. After blocking for 1 h with 2.5% BSA in PBS and 0.1% Tween 20, membranes were incubated with peroxidase-conjugated AffiniPure donkey antihuman IgG (H+L) antibody (Jackson ImmunoResearch, West Grove, PA, USA) or horseradish peroxidase (HRP)-conjugated goat anti-human IgAα chain antibody (Bethyl Laboratories, Montgomery, TX, USA) for 1 h, and then washed and revealed with Super Signal West Pico chemiluminescent substrate (ThermoFisher Scientific, Waltham, MA, USA).

#### 4.3.2. The MIAA Assay

As previously published, the MIAA assay allows testing for panels of commercially available antigens or/and lysates from EBV, herpes simplex virus 1 (HSV-1), HSV-2, cytomegalovirus (CMV), varicella zoster virus (VZV), HCV, *Helicobacter pylori* (*H. pylori*), *Toxoplasma gondii* (*T. gondii)*, and *Borrelia burgdorferi* (*B. burgdorferi)* [5,14,15]. Infectious Ag were purchased from Abcam (Cambridge, UK), Advanced Biotechnologies Inc. (Columbia, MD, USA) and ImmunoDiag (Hämeenlinna, Finland). Lysates were supplied by Advanced Biotechnologies Inc. (Columbia, MD, USA) and EastCoast Bio (North Berwick, ME, USA). The arrays consist of 8 × 8 matrices that included: (i) 13 Ag: 2 for EBV, 3 for HCV, 1 for *T. gondii,* 1 for *H. pylori,* 2 for HSV-1, 2 for HSV-2, 2 for VZV; (ii) 5 lysates: CMV, *T. gondii*, *H. pylori*, HSV-1, and HSV-2; (iii) 2 mixes: one of 5 CMV Ag, and one of 2 *B. burgdorferi* Ag. For hybridization, Ig concentrations were adjusted to 400 µg/mL for serum and from 50 to 200 µg/mL for purified monoclonal Igs. 80 µL of samples were incubated for 2 h at room temperature. After washing, slides were incubated with a labelled secondary antibody (0.4 µg/mL Dylight^TM^ 680 Labelled Goat anti-human IgG (H+L), from Sera Care, Milford, MA, USA; Ref. 5230-0342, or DyLight^TM^ 680 goat anti-human IgAα chain from ImmunoReagent, Raleigh, NC, USA; Réf. GtxHu-001-E680NHSX). Fluorescence signals, detected with the Odyssey infrared imaging system scanner at 21 μm resolution (LI-COR Biosciences, Lincoln, NE, USA) were quantified using the GenePix^®^ Pro 4 Microarray Acquisition & Analysis Software (Molecular Devices, Sunnyvale, CA, USA).

### 4.4. Analysis of IgG sialylation

Whenever possible, for each patient, samples from preparations of non-clonal Igs and of purified monoclonal Ig were studied in parallel, as previously published [15]. An ELLA (Enzyme Linked Lectin Assay) was used for IgG sialylation detection, and an ELISA (Enzyme Linked Immuno Sorbent Assay) for total IgG detection [15]. Ninety-six-well plates (Nunc MaxiSorp™) were coated overnight at 4 °C with 50 μL of Affinipure donkey anti-human IgG, Fcγ-specific fragment antibody (Jackson ImmunoResearch, West Grove, PA, USA) diluted at 1/250 (5.2 µg/mL, ELLA) and 1/1000 (1.3 µg/mL, ELISA) in 25 mM borate buffer pH 9. After 3 washes with 200 μL PBS-Tween 0.05% (Sigma, Saint Louis, MO, USA), 100 μL periodic acid (5 mM) per well were added for 10 min at room temperature, protected from light. The plates were then saturated with 100 μL of B-grade bovine gelatin (Sigma, Saint Louis, MO, USA) 0.25% in PBS-Tween 0.01%, at 37 °C, for 2 h. After 3 washes, samples were diluted in PBS-Tween 0.1% and deposited in triplicates containing 1.25 ng/well for detection of total IgG, or 100 ng/well for sialylation detection. Total IgG quantity was revealed by incubating the plates with 50 μL of peroxidase affinipure donkey anti-human IgG (H+L) diluted 1/1000 (0.8 µg/mL, Jackson ImmunoResearch, West Grove, PA, USA) for 1 h. Sialic acid was revealed using 50 μL biotinylated *Sambucus nigra* agglutinin (SNA) diluted 1/750 (2 μg/mL, Glycodiag, Orleans, France) for 90 min and then 50 μL streptavidin HRP diluted 1/1000 (1 µg/mL, Vector laboratories, Burlingame, CA, USA) for 1 h, at 37 °C. Then, 50 μL of TMB, the chromogenic substrate for HRP (Sigma-Aldrich, Saint Louis, MO, USA) was added, and the reaction was stopped by 50 μL sulfuric acid 0.5 M, after 5 min for IgG detection and after 15 min for sialic acid detection. Optical densities (OD) were measured using Spark 10 M multimode microplate reader (Tecan, Männedorf, Switzerland) at 450 nm. The relative sialylation was expressed as the sialic acid/IgG OD ratio. Control samples were used in all experimental settings to assess reproducibility.

### 4.5. Quantification of Inflammation Cytokines

Frozen aliquots of serum were used to quantify 40 cytokines and 2 soluble cytokine receptors linked to inflammation or/and infection using the Luminex technology and Bio-Plex Pro Human Cytokine Panel kits (Bio-Rad, Hercules, CA, USA), following the manufacturer’s instructions [15].

### 4.6. Statistics

Data analysis was performed by GraphPad Prism 6.01 software. Patient parameters were expressed as medians and ranges, or/and means ± standard error of the mean (SEM). The Chi-2 test was used for categorical variables. For continuous variables (n ≥ 30) the Student *t*-test (2 groups) and the one-way ANOVA followed by Tukey’s post hoc test (more than 2 groups) were used. For continuous variables (n < 30), a normality test was systematically performed for each group. In nonparametric conditions, a Mann–Whitney *t*-test (2 groups) was performed. The tests used are indicated in the legends of figures and tables. A *p*-value below 0.05 was considered statistically significant.

## 5. Conclusions

Our study shows that autoimmunity against GlcSph is frequent in the context of sporadic MGUS/SMM and MM and presumably represents the initial pathogenic event of 16% sporadic MGUS/SMM and MM cases. It is important to identify MGUS/SMM or MM cases with a GlcSph-reactive monoclonal Ig, firstly because MM patients with a GlcSph-reactive monoclonal Ig appear to have a mild form of disease, and secondly because GlcSph-reducing therapy may suppress the clonal GlcSph-reactive Ig and thus could be of interest for all patients with a GlcSph-reactive monoclonal Ig. Finally, the study highlights the importance of identifying the target of monoclonal Igs, and the necessity to analyze a patient’s monoclonal Ig separately from the non-clonal Igs.

## Figures and Tables

**Table 1:** Glucosylsphingosine (GlcSph)-reactivity of serum Igs and specificity of monoclonal Igs of monoclonal gammopathy of undetermined significance (MGUS)/smoldering multiple myeloma (SMM) and multiple myeloma (MM) patients.

Serum Ig Reactivity	Healthy Donors (*n* = 41) *	MGUS/SMM (*n* = 143) *	MM (*n* = 90)	*p* Value, MM vs. MGUS/SMM **
Serum with GlcSph-reactive immunoglobulins (Igs)	2 (4.9%)	68/138 (49.3%) ^1^	35/90 (38.9%) ^1^	*p* = 0.223
Reactivity of purified Mc Ig				
GlcSph	-	24 (16.8%)	13 (14.4%)	NS
Epstein-Barr virus (EBV) nuclear antigen-1 (EBNA-1)	-	53 (37.1%)	22 (24.4%)	*p* = 0.0608
Other pathogen of the multiplexed infectious antigen microarray (MIAA)	-	35 (24.5%)	7 (7.8%)	*p* = 0.0014
Unknown		31 (21.7%)	48 (53.3%)	*p* < 0.00001

* Due to lack of samples, the GlcSph reactivity of serum Igs was assessed for 41/46 healthy donors and 138/143 MGUS/SMM patients. ^1^ *p* < 0.00001 vs. healthy donors, Fisher exact test. ** MM vs. MGUS/SMM using the Fisher exact test, *p* < 0.05 was considered significant. Mc Ig = purified monoclonal Ig. NS: not significant.

**Table 2:** Infectious pathogen-specificity of monoclonal Igs in MGUS/SMM and MM according to the presence or absence of non-clonal GlcSph-reactive Igs.

Patients *	Without GlcSph-Reactive Igs	With Non-Clonal GlcSph-Reactive Igs *	*p* Value
**MGUS/SMM**	*n* = 75	*n* = 44	–
EBV (EBNA-1)	37 (49.3%) ^1^	16 (36.4%)	NS
Other pathogen of the MIAA	17 (22.7%) ^2^	18 (40.9%) ^4^	*p* = 0.0398 ^6^
Unknown	21 (28.0%) ^3^	10 (22.7%) ^5^	NS
**MM**	*n* = 55	*n* = 22	–
EBV (EBNA-1)	15 (27.3%)	7 (31.8%)	NS
Other pathogen of the MIAA	4 (7.3%)	3 (13.6%)	NS
Unknown	36 (65.4%)	12 (54.5%)	NS

* This analysis was performed without the 37 patients (24 MGUS, 13 MM) who presented with a GlcSph-reactive monoclonal Ig. Significant differences observed using the Fisher exact test: ^1^ *p* = 0.0121, ^2^ *p* = 0.0282 and ^3^ *p* < 0.0001, MGUS/SMM vs. MM without GlcSph-reactive Igs; ^4^ *p* = 0.0466 and ^5^ *p* = 0.0108, MGUS/SMM vs. MM with non-clonal GlcSph-reactive Igs; ^6^ MGUS/SMM patients with vs. without GlcSph-reactive Igs. MIAA: multiplex infectious antigen microarray. NS: not significant.

**Table 3:** Characteristics of MM patients with GlcSph-reactive Ig(s).

Characteristics of MM Patients	No GlcSph-Reactive Ig (*n* = 55)	With GlcSph-Reactive Igs (*n* = 35)	Analysis According to Mc Ig Specificity		
			GlcSph (*n* = 13)	EBV EBNA-1 (*n* = 22)	Unknown (*n* = 45)
**Sex**					
Nbr	55	34	13	22	45
M/F (male %)	27/28 (49.1%)	23/12 (65.7%)	7/6 (53.8%)	12/10 (54.5%)	24/21 (53.3%)
**Age at diagnosis (yrs)**					
Nbr	53	35	13	22	41
Median	64.0	69.7	61.0	61.1	64.5
Range (Min-Max)	41–90	43–90	54–80	46–87	41–90
**Amount of Mc Ig (g/L)**					
Nbr	55	33	13	22	42
Median	20.0	27.0	24.0	27.0	22.9
Range (Min-Max)	8.5–68	4.0–68	11–68	11–59	4–55
**BM plasma cells (%)**					
Nbr	41	27	10	18	33
Median	16.0	26.5	35.5	32.5	16.0
Range (Min-Max)	1–93	1–98	11–65	2–98	1–89
**β_2_-microglobulin (mg/L)**					
Nbr	29	19	9	22	21
Median	3.1	3.9	2.5	4.1	3.1
Range (Min-Max)	1.3–14.0	1.9–12.1	1.9–12.1	1.3–16.0	1.5–14.0
Nbr > 3.5 mg/L	11 (37.9%)	10 (55%)	4 (44.4%)	13 (59.1%)	8 (38.1%)
**Bone lesions**					
Nbr	52	34	13	22	43
Nbr with lesions (%)	38 (73.1%)	21 (61.8%)	7 (53.8%) ^1^	16 (72.7%)	30 (69.7%)
**ISS Stage**					
Nbr	30	20	8	16	19
Stage I	16 (53.3%)	10 (50%)	5 (62.5%)	6 (37.4%)	11 (57.9%)
Stage II	6 (20.0%)	4 (20%)	2 (25.0%)	3 (18.8%)	5 (26.3%)
Stage III (%)	8 (26.7%)	6 (30%)	1 (12.5%)	7 (43.8%)	3 (15.8%)
**DSS Stage**					
Nbr	45	28	13	22	34
Stage I	11 (24.4%)	12 (42.9%)	7 (53.8%)	6 (27.3%)	10 (29.4%)
Stage II	12 (26.7%)	6 (21.4%)	4 (30.8%)	3 (13.6)	12 (35.3)
Stage III (%)	22 (48.9%)	10 (35.7%)	2 (15.4%) ^2^	13 (59.1%)	12 (35.3%)

Nbr: number of patients; Mc Ig = purified monoclonal Ig; BM = bone marrow. Statistics were performed using the Chi-2 test for categorical variables and the Mann–Whitney test for continuous variables. Significant differences are indicated: ^1^ *p* = 0.0286 and ^2^ *p* = 0.0158 vs. MM patients with EBNA-1-reactive monoclonal Ig, Fisher exact test.

## Supplementary Materials

The following are available online at https://www.mdpi.com/2072-6694/12/5/1254/s1, Figure S1: Representation of the numbers of MGUS/SMM and MM patients for whom the four different assays used in the study were performed, Figure S2: Results of GlcSph assays for additional MGUS patients, Figure S3: Results of GlcSph assays for healthy donors, Table S1: Characteristics of MGUS/SMM and MM patients, Table S2: Characteristics of MGUS/SMM patients with GlcSph-reactive Ig(s) in serum, Table S3: List of cytokines, chemokines and receptors quantified in the serum of patients, and values observed in healthy donors.
