# Supplementary material for: Characteristics of MGUS and Multiple Myeloma According to the Target of Monoclonal Immunoglobulins, Glucosylsphingosine, or Epstein-Barr Virus EBNA-1
Source: Cancers (Basel). 2020 May 15;12(5):1254. doi: 10.3390/cancers12051254 (PMC7281552; doi:10.3390/cancers12051254)
Supplement: Supplementary file 1 [file cancers-12-01254-s001.pdf]

cancers

Article

Characteristics of MGUS and Multiple Myeloma According to the Target of Monoclonal Immunoglobulins, Glucosylsphingosine or Epstein-Barr Virus EBNA-1

Adrien Bosseboeuf, Nicolas Mennesson, Sophie Allain-Maillet, Anne Tallet, Eric Piver, Olivier Decaux, Caroline Moreau, Philippe Moreau, Philippe Lehours, Francis Mégraud, Valéry Salle, Edith Bigot-Corbel, Jean Harb, and Sylvie Hermouet

Supplementary Materials.

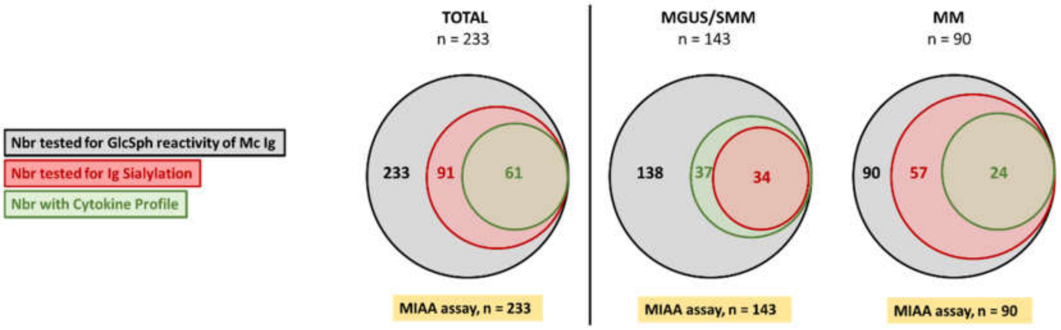

Figure S1. Representation of the numbers of MGUS/SMM and MM patients for whom the four different assays used in the study were performed.

Four assays were used in this retrospective assay: the MIAA assay (performed for all patients included in this study), the GlcSph assay (performed for all but 5 patients), the Ig sialylation assay and multiplexed cytokine quantification (performed for fractions of patients, as indicated).

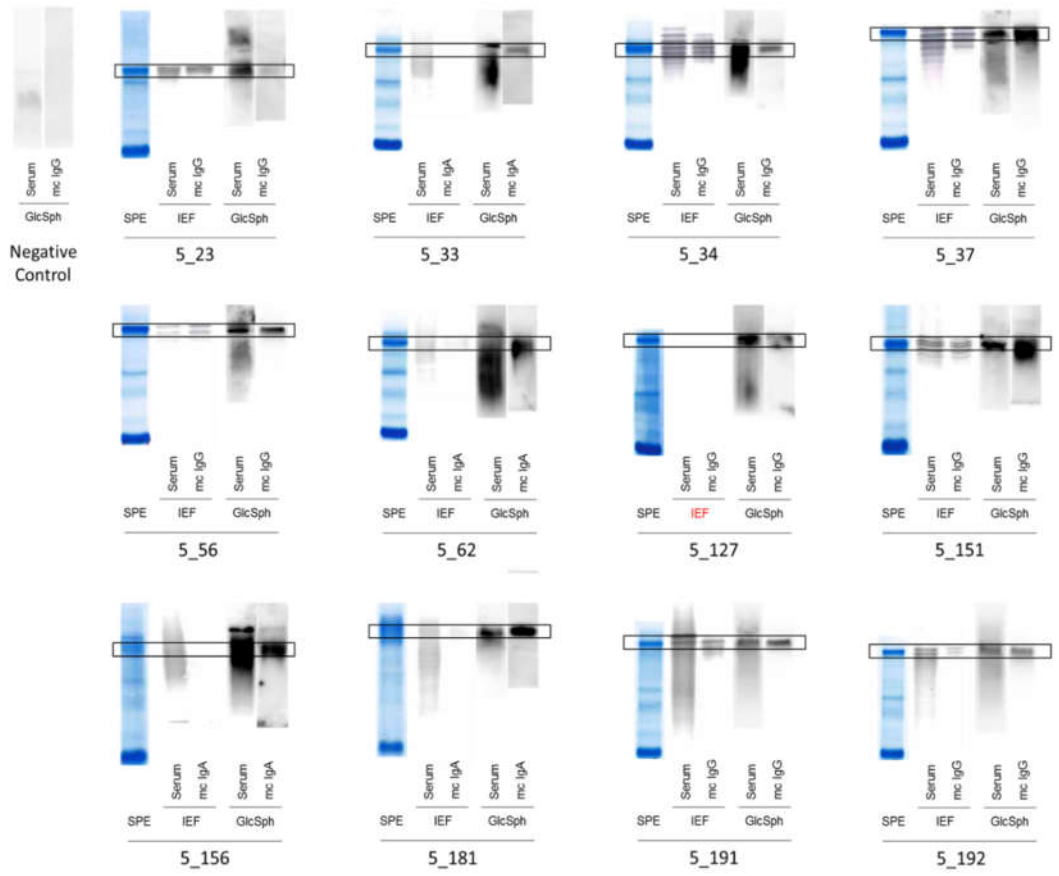

Figure 2. Results of GlcSph assays for additional MGUS patients.

Results of the analysis of GlcSph-reactivity of serum Igs and monoclonal Igs obtained for 12 additional MGUS patients: Measurement of Ig concentration, separation of monoclonal Igs from other Igs, and verification of purity are performed as published [5–7,14,15]. Signals corresponding to the patient’s monoclonal Ig are encircled. The negative control is a patient with no GlcSph-reactive Ig in serum. SPE = Serum protein electrophoresis; Mc Ig = purified monoclonal Ig.

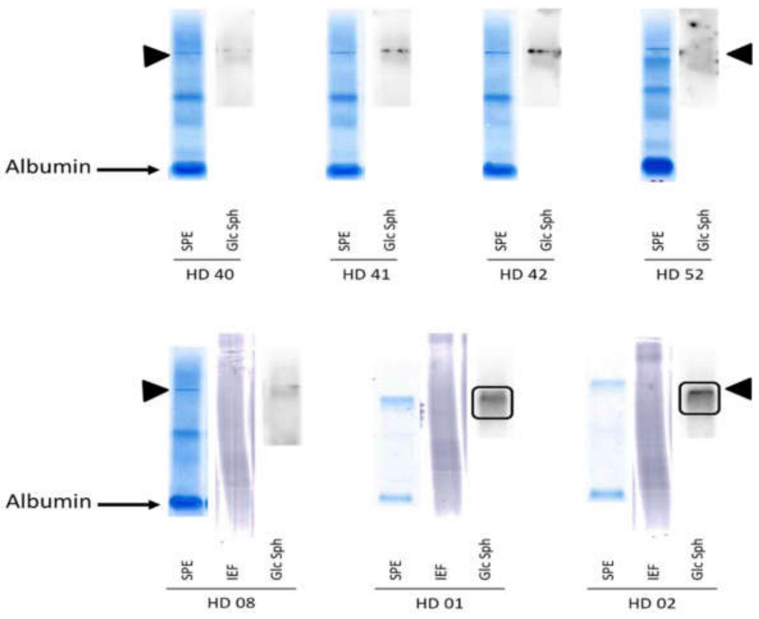

Figure S3. Results of GlcSph assays for healthy donors.

Examples of results of the GlcSph assay obtained for serum Igs from healthy donors: After measurement of Ig concentration, GlcSph-specific immunoblotting was perfomed as described in Methods. Images of the SPE and GlcSph immunoblot are shown in parallel; black arrows indicate the line of sample deposit. Forty-one healthy donors were tested, and 39 were found to be negative for GlcSph-reactive Igs ; the results of donors HD40, HD41, HD52 and HD08 are shown as examples of serum with no GlcSph-reactive Igs. The presence of polyclonal GlcSph-reactive Igs was detected for 2 healthy donors (HD01, HD02); the signals corresponding to GlcSph-reactive Igs are encircled. IEF were performed for patients HD08, HD01, and HD02, which confirmed the absence of monoclonal Ig in the serum of these individuals.

Table S1. Characteristics of MGUS/SMM and MM patients.

Patient Parameters	Healthy Donors*	MGUS/SMM*	MM
Nbr of patients (n)	41	143	90
Sex (M/F) (Male %)	ND	60/45 (57.1%)	50/40 (54.5%)
Age (Years)			
Median	ND	68.1	67.0
Range (Min-Max)	ND	31–95	42–92
Monoclonal Ig (g/L)			
Median	NA	16.0	23.0
Range (Min-Max)	NA	4.0–39.8	4.0–68.0
β_2-microglobulin (mg/L)			
Median	ND	2.4	3.1
Range (Min-Max)	ND	1.1–10.1	1.3–14.0
Bone lesions (%)	NA	5/68 (7.4%)	59/86 (68.6%)
Leukocytes ($\times 10^9$/L)			
Median	ND	7.1	5.2
Range (Min-Max)	ND	3.3–16.0	1.3–19.0
Hemoglobin (g/dL)			
Median	ND	13.3	10.7
Range (Min-Max)	ND	7.6–16.9	5.4–15.5
Platelets ($\times 10^9$/L)			
Median	ND	225.0	199.5
Range (Min-Max)	ND	75.0–580.0	15.0–529.0
ISS Stage III (%)	NA	NA	14 (28.0%)
DSS Stage III (%)	NA	NA	32 (43.8%)

*For healthy donors and certain MGUS patients, biological and clinical information was not available or partial. Cytogenetics data were not available for most MM patients. Nbr = number; ND: no data; NA: not applicable.

Table S2. Characteristics of MGUS/SMM patients with GlcSph-reactive Ig(s) in serum.

MGUS/SMM Patients	Without GlcSph-Reactive Ig(s) (*n* = 75)	With GlcSph-reactive Non-clonal Ig(s) (*n* = 68)	With GlcSph-Reactive Mc Ig (*n* = 24)	With EBV EBNA-1-Reactive Mc Ig (*n* = 53)
Sex				
Nbr	63	65	24	49
M/F (Male %)	36/27 (57.1%)	37/28 (56.9%)	11/13 (45.8%)	34/15 (69.4%) [1]
Age at diagnosis (yrs)				
Nbr	63	65	24	49
Median	67.6	68.7	73.5	69.8
Range (Min-Max)	41–95	31–89	53–89	31–89
Amount of Mc Ig (g/L)				
Nbr	68	67	24	53
Median	17.2	13.0 [2]	15.0	16.7
Range (Min-Max)	4.5–39.8	4.0–38.4	6.0–29.0	4.5–33.5

Nbr: number; Mc Ig = purified monoclonal Ig. [1] $p = 0.0294$ vs MGUS/SMM patients without EBNA-1-reactive Mc Ig, Fisher exact test; [2] $p = 0.0123$ vs MGUS/SMM patients without GlcSph-reactive Igs, Mann Whitney test.

Table S3. List of cytokines, chemokines and receptors quantified in the serum of patients, and values observed in healthy donors.

Molecules.	Healthy Donors (*n* = 9)	
	**Median (pg/mL)**	**Range (pg/mL)**
IL-4	4.50	3.61-5.15
IL-10	0.81	ND-165.5
IL-11	ND	ND-ND
IL-13	1.19	0.05-2.84
IL-1β	1.32	0.62-1.53
IL-1Rα	73.28	13.52-128.28
IL-2	ND	ND-ND
IL-2Rα	69.81	ND-162.12
IL-6	ND	ND-7.56
IL-7	0.62	ND-10.57
IL-8	17.92	5.26-43.15
IL-9	59.15	42.62-96.80
IL-12(p70)	15.91	11.09-35.49
IL-15	ND	ND-ND
IL-23	ND	ND-2.77
IFN-α2	ND	ND-2.89
IFN-γ	30.91	17.23-34.72
TNF-α	32.68	25.75-38.67
MIP-1α	2.50	0.98-4.96
G-CSF	17.67	9.49-24.46
GM-CSF	ND	ND-ND
FGF basic	45.15	ND-70.39
HGF	302.57	125.14-471.16
PDGF-bb	543.07	271.84-666.48
TGF-β1	16764.8	4928.0-47560.0
TGF-β2	2553.75	1512.50-3078.00
TGF-β3	441.60	300.00-789.60
IL-5	ND	ND-ND
IP-10	692.68	482.47-1392.16
LIF	ND	ND-4.52
VEGF	ND	ND-13.53
RANTES	11582.3	9480.4-14531.4
SDF-1α	750.42	609.70-969.86
Eotaxin	141.24	91.56-318.96
MIG	325.32	210.74-1192.63
MCP-1 (MCAF)	ND	ND-ND
MIP-1β	421.48	310.31-726.69
IL-17	216.39	124.15-254.54
IL-22	ND	ND-ND
IL-26	ND	ND-1.71
IL-33	0.27	ND-3.46
Leptin, Males	2200.37	583.36-4860.99
Leptin, Females	7102.62	4005.0-10200.2

Molecules in blue were found to be significantly elevated in MGUS and MM compared to healthy donors [15]. The values obtained for healthy donors, previously published [15], are provided for information. ND: not detectable (below detection level).
